# Neurosurgery in Costa Rica: historical evolution and contemporary state

**DOI:** 10.3389/fsurg.2026.1890845

**Published:** 2026-10-29

**Authors:** Miriam Acuña-Quirós, Tze-Yu Yeh-Hsieh, Thomas M. Freiman, Florian Geßler, Silvia Hernández-Durán

**Affiliations:** 1 Department of Neurosurgery, Hospital México, Caja Costarricense del Seguro Social, San José, Costa Rica; 2 Facultad de Medicina, Universidad de Costa Rica, San Pedro de Montes de Oca, San José, Costa Rica; 3 Department of Neurosurgery, Rostock University Medical Center, Rostock, Germany

**Keywords:** Costa Rica (Central America), history of neurosurgery, international collaboration, Latin America, neurosurgical workforce

## Abstract

**Background:**

The development of neurosurgery in Central America has been shaped by the gradual expansion of public healthcare systems, academic medical training, and international collaboration. Costa Rica represents a distinctive case within Latin America due to its universal healthcare model and centralized hospital network. This study aims to describe the origins, institutional development, and contemporary status of neurosurgery in Costa Rica within the broader context of Latin American neurosurgical history.

**Methods:**

This is a historical and descriptive narrative review, not a systematic review; no protocol was registered. Sources included institutional reports and archives from the Caja Costarricense de Seguro Social and the University of Costa Rica, national and international public-health statistics, English- and Spanish-language peer-reviewed literature, and biographical and historiographic material.

**Results:**

The emergence of neurosurgery in Costa Rica occurred during the mid-twentieth century alongside the expansion of the national public healthcare system. Early neurosurgical procedures were initially performed by general surgeons until the return of internationally trained physicians established dedicated neurosurgical services. During the 1960s and 1970s, neurosurgical departments were established in the country's principal tertiary hospitals. Today, approximately 54 neurosurgeons provide care to a population of over five million inhabitants through a centralized national referral network. Modern practice incorporates advanced technologies such as neuronavigation and intraoperative neuromonitoring. Nevertheless, challenges persist, including workforce limitations, growing demand for neurosurgical care, and the absence of domestic subspecialty training programs and academic output.

**Conclusion:**

The history of neurosurgery in Costa Rica illustrates how specialized surgical disciplines can emerge within a small but highly organized healthcare system through academic development and international collaboration. Continued investment in training, infrastructure, and global partnerships will be essential to address current challenges and support the future growth of neurosurgery in the country.

## Introduction

The development of neurosurgery in Costa Rica reflects a broader narrative of medical modernization in Latin America, characterized by the gradual institutionalization of specialized care, the influence of international training networks, and the integration of neurosurgical practice into national public health systems. Although modern neurosurgery emerged as a distinct surgical discipline in the early twentieth century in North America and Europe (1–4), its adoption in Central America occurred more gradually, shaped by local educational infrastructures, healthcare policy, and the availability of specialized technology (5–7). In Costa Rica, the emergence of neurosurgery represents a convergence of these factors, illustrating how a small nation built a sophisticated neurosurgical tradition within a relatively short period of time.

Despite the significance of these developments, the historical evolution of neurosurgery in Costa Rica has received limited scholarly attention. Reconstructing this history provides insight not only into the formation of a national neurosurgical tradition but also into the mechanisms through which specialized medical knowledge spreads and adapts to different institutional and healthcare contexts, emphasizing the importance of diversification of training. This study examines the origins and development of neurosurgery in Costa Rica, highlighting the establishment of neurosurgical services, the role of early pioneers, and the institutional and educational structures that supported the consolidation of the field. By situating these developments within the wider trajectory of neurosurgical progress in Latin America, this paper contributes to a more comprehensive understanding of the global history of neurosurgery.

## Methods

This manuscript presents a historical and descriptive narrative review of the origins and contemporary status of neurosurgery in Costa Rica; it was not conducted as a systematic review, and no review protocol was registered. Sources were identified through searches conducted between September 2025 and January 2026 and comprised four categories: (1) institutional reports and internal historiographic material from the Caja Costarricense de Seguro Social (CCSS) and its library (BINASS), and the Faculty of Medicine of the University of Costa Rica (UCR); (2) governmental and international public-health statistics, including the CEPALSTAT Statistical Yearbook for Latin America and the Caribbean and World Health Organization publications (8); (3) peer-reviewed literature retrieved from PubMed/MEDLINE, SciELO, and Google Scholar using combinations of the terms “neurosurgery,” “Costa Rica,” “Central America,” “history of medicine,” “workforce,” and “residency training,” with no lower date limit given the historical scope of the topic, but restricted to sources published in English or Spanish; and (4) biographical and archival material, including institutional anniversary volumes, hospital historical reviews, and specialty-society records. Reference lists of identified sources were additionally hand-searched for further relevant material.

Where possible, historical claims, including institutional founding dates and biographical details of early neurosurgeons, were cross-checked against at least two independent sources (for example, an institutional record together with a published historical account). Contemporary workforce figures were obtained directly from the registry of the Colegio de Médicos y Cirujanos de Costa Rica (accessed January 2026) and cross-referenced against CCSS institutional reports. No unpublished patient-level or confidential institutional data were used. Consistent with the descriptive and historical nature of this review, no formal quality appraisal or risk-of-bias assessment was performed; the resulting limitations of this approach are discussed below.

### Costa Rican healthcare system

Costa Rica, a Central American nation with an estimated population of approximately 5.18 million inhabitants, has long been recognized as one of the strongest health performers in Latin America. Current indicators reflect this performance, with a life expectancy at birth of 77.3 years and an infant mortality rate of 6.8 per 1,000 live births (8). Physician density is estimated at 27.7 physicians per 10,000 inhabitants, placing the country above the regional average and reflecting the strength of its public health infrastructure (9). These outcomes are frequently attributed to Costa Rica's universal public healthcare model, which is built upon constitutional principles of universality, solidarity, and mandatory contribution (10).

A pivotal milestone in the formation of this system occurred in 1941 with the creation of the Costa Rican Social Security Fund (CCSS) under the administration of Dr. Rafael Ángel Calderón Guardia (11). The CCSS was conceived as a social insurance institution designed to provide universal healthcare access while integrating health financing and service delivery within a single administrative structure. The 1943 constitutional reforms granted the CCSS institutional autonomy, insulating healthcare governance from political fluctuations and allowing long-term technical planning (12). In contrast to many Latin American healthcare systems characterized by fragmentation between public, private, and social insurance sectors, Costa Rica developed a relatively unified model that facilitated the equitable expansion of hospital services and specialized medical care (10, 13).

The foundations of modern Costa Rican healthcare, however, predate the twentieth century. Institutional medical practice began to take shape during the nineteenth century with the establishment of Hospital San Juan de Dios in 1845, one of the oldest hospitals in Central America (14). The creation of the Protomedicato in 1857 introduced regulatory oversight for medical practice and represented an early effort to standardize professional qualifications within the country. These developments laid the groundwork for the later evolution of specialized medicine and the expansion of hospital-based clinical training (15). Similar processes occurred throughout Latin America during this period, as countries gradually transitioned from colonial medical traditions toward modern hospital-centered healthcare systems influenced by European medical models (16).

Within this broader regional transformation, the gradual strengthening of Costa Rica's hospital network during the twentieth century created the institutional conditions necessary for the emergence of highly specialized surgical disciplines, including neurosurgery.

### Neurosurgery in Costa Rica: the first era

The emergence of neurosurgery as a distinct discipline in Costa Rica occurred during the mid-twentieth century, mirroring developments across Latin America where the specialty began to consolidate between the 1940s and 1970s. Prior to this period, neurosurgical procedures were generally performed by general surgeons with an interest in trauma or neurological pathology, as formal neurosurgical training programs were largely unavailable within the region (17, 18). The establishment of the Faculty of Medicine at the University of Costa Rica (UCR) in 1961 provided the essential academic framework for formalized neurosurgical training (19).

A seminal figure in this transition was Dr. Eduardo Guevara Coronado, who, after obtaining his medical degree in Spain, completed his neurosurgical residency at Johns Hopkins University (18). Upon returning to Costa Rica in 1963, Guevara Coronado became the first physician officially registered as a neurosurgeon by the College of Physicians and Surgeons of Costa Rica. In addition to serving as the inaugural Professor of Neurosurgery at the UCR, he founded the Neurosurgical Service at the National Children's Hospital (*Hospital Nacional de Niños*) (20). His leadership was instrumental in pioneering specialized pediatric neurocare and establishing protocols for the surgical management of complex congenital malformations, such as spinal dysraphism (15, 18, 20).

While Guevara Coronado focused on pediatric care, other visionary surgeons established services for the adult population. In the early 1960s, the Hospital San Juan de Dios became the epicenter for adult neurosurgical development. Practitioners transitioning from general surgery began implementing specialized techniques for cranial trauma and hypertensive hemorrhages (14, 15, 18). The inauguration of Hospital México in 1969 represented a further structural shift; conceptualized as a high-complexity facility, it assumed the role of the national reference center for neurotrauma and vascular pathologies at the time (21, 22). This period saw the influence of specialists such as Dr. Mario Esquivel Monge and Dr. Julián Sedó, whose international training was pivotal in introducing the first surgical microscopes. This facilitated a paradigm shift toward microsurgical precision, establishing the foundations for the management of intracranial aneurysms and complex skull-base tumors (20).

By the mid-1970s, the Hospital Rafael Ángel Calderón Guardia inaugurated its own neurosurgical department, effectively completing the three main hospitals of specialized neurological care within the public social security network (18). This institutional expansion allowed for a strategic distribution of clinical labor: while the National Children's Hospital focused on congenital malformations, adult hospitals began to sub-specialize in spinal surgery and neuro-oncology (17, 18, 20).

The academic structure supporting this clinical development was closely linked to the UCR, specifically its Faculty of Medicine and the postgraduate programs in medical specialties. Residency training in Costa Rica emerged through a cooperative framework between the UCR and the CCSS. Clinical training occurred in CCSS hospitals, while academic oversight—including curriculum design, evaluation standards, and formal accreditation of specialties—was provided by the university, linking hospital-based training with an academic postgraduate education system, ensuring that neurosurgical training aligned with international benchmarks (19, 23, 24).

This model differs in important ways from residency structures in other regions. In the United States, neurosurgical residency programs are typically organized within university-affiliated teaching hospitals and accredited nationally through bodies such as the Accreditation Council for Graduate Medical Education, with strong integration between academic departments and hospital training (25, 26). In much of continental Europe, specialist training is primarily regulated by professional medical chambers through formal training regulations, while hospitals and department heads supervise day-to-day training and universities play a more limited role after undergraduate medical education (27–31). Costa Rica thus represents a hybrid institutional model in which neurosurgical specialization is formally embedded within a university postgraduate program while clinical training takes place within a centralized public hospital network. This arrangement helped standardize training during the formative decades of neurosurgery in the country and contributed to the consolidation of specialized services across the national healthcare system. The historical milestones of Costa Rican neurosurgery are summarized in Table 1.

**Table 1:** Key milestones in the development of Costa Rican neurosurgery.

Year	Milestone
1845	Hospital San Juan de Dios founded, among the oldest hospitals in Central America
1857	Creation of the Protomedicato, introducing early regulatory oversight of medical practice
1941	Founding of the Caja Costarricense de Seguro Social (CCSS)
1943	Constitutional reform grants the CCSS institutional autonomy
1961	Establishment of the Faculty of Medicine at the University of Costa Rica (UCR)
1963	Dr. Eduardo Guevara Coronado returns from training at Johns Hopkins; becomes the first physician officially registered as a neurosurgeon in Costa Rica; founds the Neurosurgical Service at the Hospital Nacional de Niños
Early 1960s	Adult neurosurgical services begin developing at Hospital San Juan de Dios
1969	Hospital México inaugurated as the national referral center for neurotrauma and vascular pathology
Mid-1970s	Hospital Rafael Ángel Calderón Guardia establishes its neurosurgical department, completing the three main CCSS neurosurgical centers

### Present era

In the twenty-first century, neurosurgery in Costa Rica has entered a phase characterized by technological modernization and institutional consolidation within the national healthcare system. Clinical neurosurgical services are primarily delivered through the hospital network of the CCSS, where Hospital México, Hospital San Juan de Dios, and Hospital Rafael Ángel Calderón Guardia function as the principal referral centers for adult neurosurgical care, while the Hospital Nacional de Niños remains the national center for pediatric neurosurgery. This network reflects the broader structure of Costa Rica's universal healthcare system, which concentrates highly specialized services within tertiary hospitals in the capital while maintaining nationwide referral pathways through regional facilities.

The neurosurgical workforce in Costa Rica currently consists of approximately 54 practicing specialists registered with the Colegio de Médicos y Cirujanos de Costa Rica, a relatively small but highly trained professional community responsible for providing neurosurgical care to the entire national population (32). Although the specialty remains historically male-dominated, gradual demographic changes are occurring. Women currently represent 15% (8/54) of the neurosurgical workforce, reflecting broader global patterns in neurosurgical gender representation (6, 33, 34). With a national population of roughly 5.18 million inhabitants, this corresponds to approximately 1.05 neurosurgeons per 100,000 population (or one neurosurgeon per ∼95,000 inhabitants) (Figure 1). In global terms, this density places Costa Rica close to widely cited benchmarks for adequate neurosurgical coverage. Historically, health policy discussions suggested one neurosurgeon per 100,000 inhabitants as a reasonable target for population coverage, while more recent estimates from global neurosurgery initiatives propose a minimum of one per 200,000 as a baseline requirement (35).

**Figure 1:**
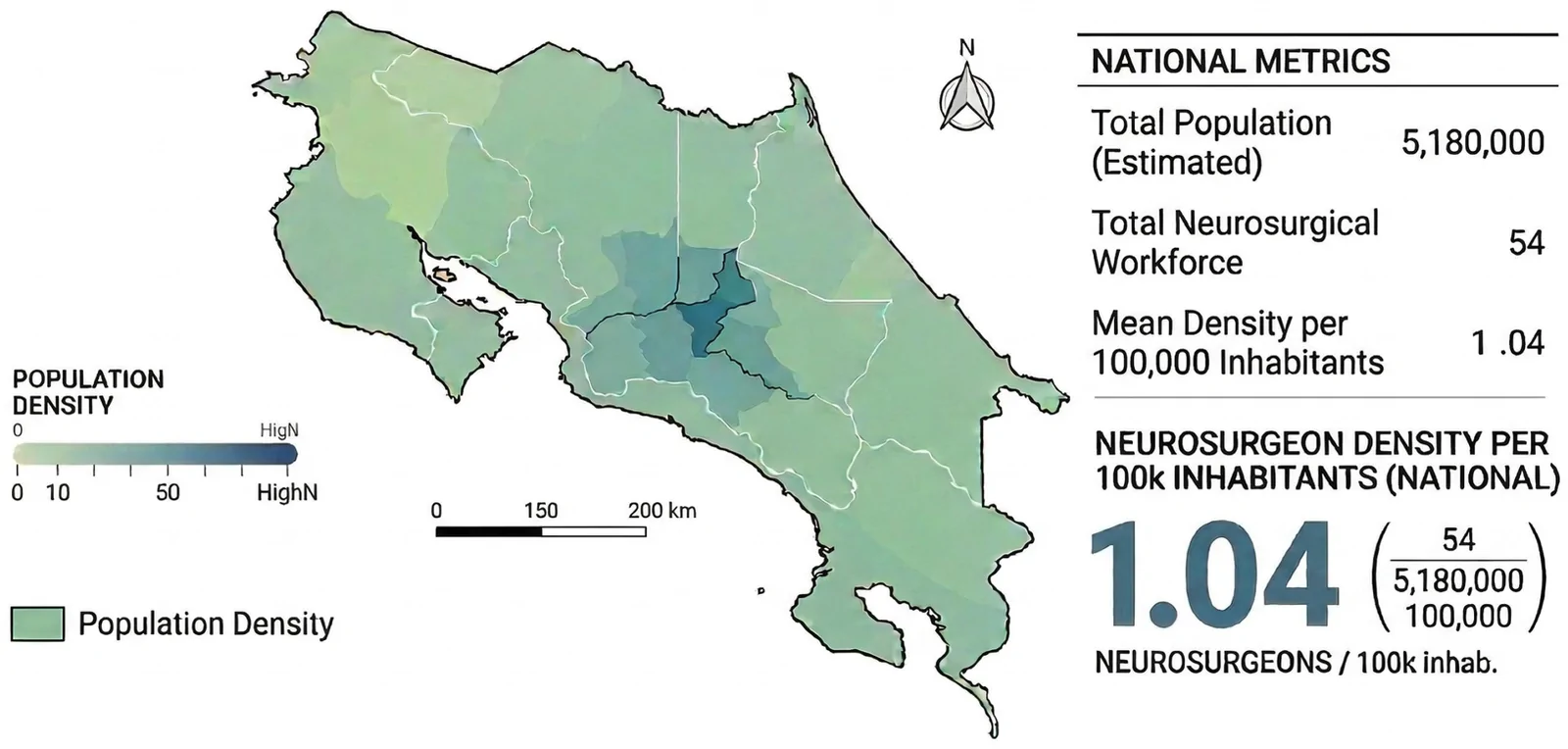
Neurosurgical workforce and population density in Costa Rica in 2026. Workforce counts were obtained from the registry of the Colegio de Médicos y Cirujanos de Costa Rica; population estimates are based on national census.

Within the country, the national workforce ratio reported above, however, masks a marked geographic concentration. All four of Costa Rica's public neurosurgical departments — at Hospital México, Hospital San Juan de Dios, Hospital Rafael Ángel Calderón Guardia, and the Hospital Nacional de Niños — are located within the *Gran Área Metropolitana* (GAM), the roughly 2,044 km^2^ urban corridor surrounding the capital city San José that concentrates a large share of the national population. CCSS regional hospitals serving outlying provinces, including Puntarenas, Guanacaste, Limón, the Northern Zone (San Carlos), and the Southern Zone (Pérez Zeledón, San Vito, Golfito), do not maintain dedicated in-house neurosurgical services and neurosurgical trauma is served by general surgeons. Patients requiring subacute or elective neurosurgical evaluation or surgery are referred from these regional centers to the GAM-based hospitals.

This concentration of neurosurgical services reflects a broader pattern observed throughout Latin America, where neurosurgeons and specialized critical care infrastructure are predominantly concentrated in metropolitan centers. These disparities are compounded by limited workforce-retention strategies and health systems that may lack the infrastructure and working conditions needed to sustain neurosurgical services outside major urban centers (33). Such centralization can increase transfer times and may contribute to poorer outcomes among patients living in remote or geographically underserved areas (5, 36, 37). At the global level, persistent inequities and centralization in neurosurgical education have been described, with training opportunities disproportionately concentrated in well-resourced metropolitan settings (38). Taken together, these findings underscore that improving the distribution of neurosurgical care—both within Costa Rica and across the region—remains an important challenge for achieving more equitable access to specialized care (5, 36, 37). Contemporary neurosurgical practice in Costa Rica incorporates advanced technologies such as intraoperative neuromonitoring, neuronavigation systems, high-resolution neuroimaging, and minimally invasive endovascular approaches (15) (Figure 2). However, despite these technological advancements, Costa Rican neurosurgery continues to face several structural and systemic challenges. One of the most significant limitations is the relatively small number of practicing neurosurgeons relative to the growing demand for specialized neurological care, particularly as the country undergoes demographic aging and experiences increasing prevalence of degenerative spinal disease, cerebrovascular pathology, and neuro-oncological conditions. These epidemiological transitions mirror trends observed across Latin America, where improvements in life expectancy have shifted the burden of disease toward chronic and complex neurological disorders (16, 33). In Costa Rica specifically, patients with non-emergent conditions such as degenerative disc disease can wait up to a year for definitive surgery because of competing patient load and operating-room priorities at the centralized referral hospitals (5).

**Figure 2:**
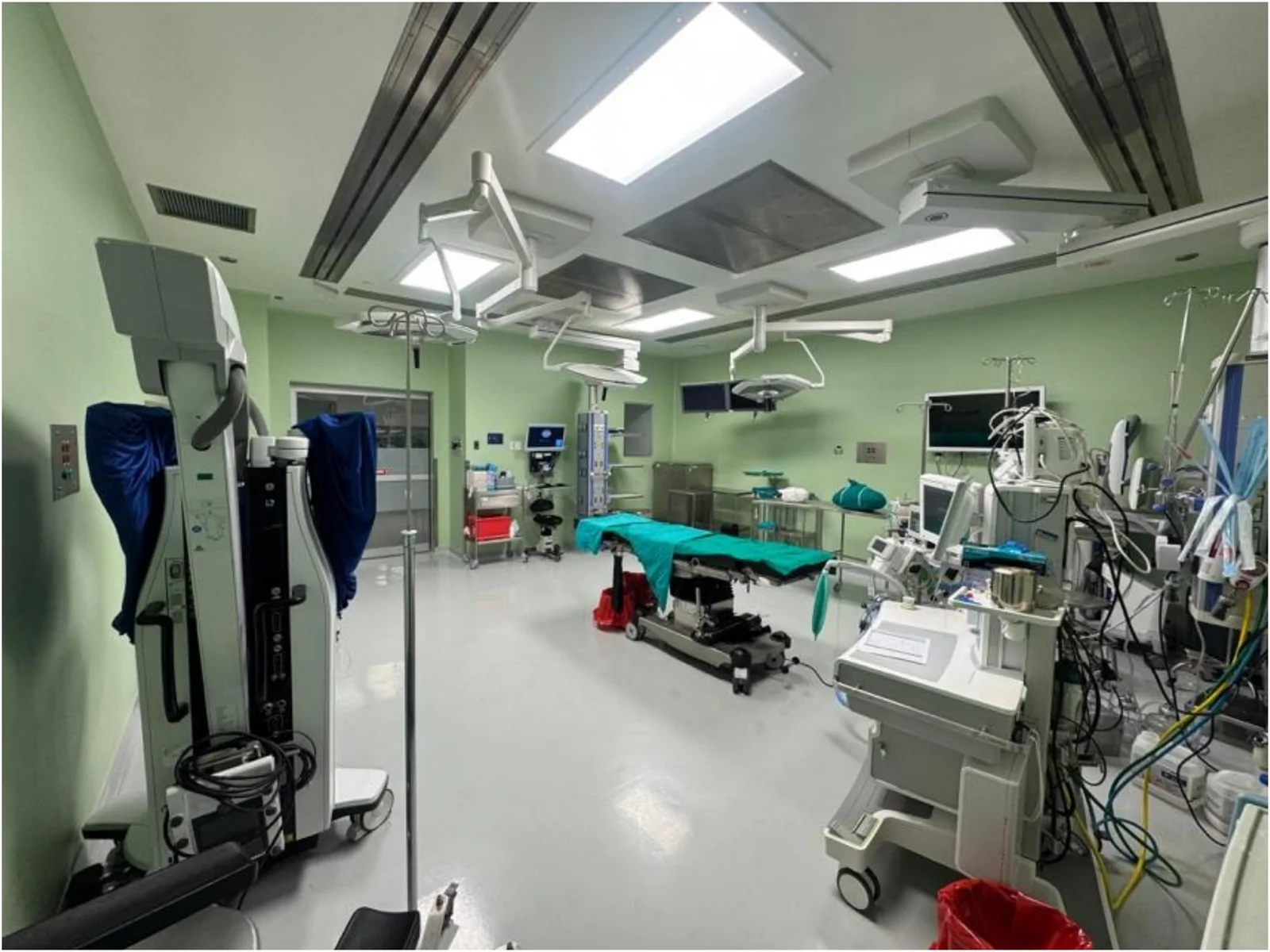
Neurosurgical operating room at the national healthcare system (Caja Costarricense del Seguro Social) in 2025. Photograph provided by the authors from their home institution; written institutional permission for publication has been obtained.

Another major challenge involves the development of subspecialty training programs. At present, Costa Rica does not maintain nationally accredited fellowships in advanced neurosurgical subspecialties such as vascular neurosurgery, complex spine surgery, skull-base surgery, or advanced pediatric neurosurgery. As a result, physicians seeking additional training must pursue fellowships abroad (39). While this international training tradition has historically played a crucial role in the development of the specialty, it also introduces limitations related to workforce planning, resource allocation, and the uneven availability of subspecialized services within the national healthcare system. This situation is not unique to Costa Rica. Across Latin America, many countries have relied heavily on international academic partnerships to train neurosurgical subspecialists. Historically, physicians from the region frequently sought training in major centers in the United States, Canada, and Europe before returning to establish modern neurosurgical services in their home countries (5, 33, 40).

In recent decades, new forms of structured international collaboration have emerged to address disparities in training opportunities and access to advanced technology. Exchange programs, visiting fellowships, and collaborative research networks have become important mechanisms for strengthening neurosurgical education in countries with limited subspecialty training infrastructure. Within this context, Costa Rica has developed new initiatives aimed at expanding the international exposure of neurosurgical residents. One such effort is a clinical rotation program established in collaboration with University of Rostock in Germany. Through this partnership, residents are able to participate in advanced neurosurgical training environments, gaining experience in highly specialized techniques such as complex spine procedures, skull-base surgery, and functional neurosurgery. Similar partnerships have been implemented in several Latin American countries, including programs linking neurosurgical centers in Brazil, Chile, and Colombia with European academic institutions. These initiatives facilitate the transfer of knowledge, promote exposure to cutting-edge surgical technologies, and strengthen international academic networks that contribute to the development of neurosurgery in the region.

However, these partnerships are not without risks, and their benefits should not be assumed to be symmetric. Partnerships between high-income and lower-resource institutions have been critiqued elsewhere in the global neurosurgery literature for tendencies toward unequal authorship credit, limited leadership roles for host-country trainees and faculty, and a structural dependency in which advanced training and technology remain concentrated in high-income partner institutions rather than being durably transferred to the local system (38, 41). On the other hand, while this pattern can provide specialized training, it can also lead to a broader phenomenon described across the region as a “neurosurgical exodus”, in which the emigration of trained specialists represents a loss of accumulated public investment (39).

There is an inherent risk that international rotations, whether by design or in practice, contribute to the same outward workforce migration described above if participating trainees do not return, or if partnerships are not paired with deliberate strategies to retain and reintegrate returning specialists into national practice (33, 39, 42). A more sustainable model would frame these collaborations explicitly as bidirectional capacity building, with shared authorship, co-developed curricula, defined pathways for trainees to return to and lead within the national system, and reciprocal exchange in which Costa Rican faculty also contribute teaching and expertise to partner institutions, rather than as a one-directional transfer of knowledge from high-resource to lower-resource settings.

Workforce sustainability also includes the working environment in which neurosurgeons and trainees practice. Recent multinational research involving Latin American neurosurgical residents has identified prolonged working hours, variable access to educational resources, limited structured mentorship, and substantial indicators of burnout and psychological strain (43). These findings are relevant to Costa Rica because a small national workforce may be particularly vulnerable to the effects of workload concentration, limited redundancy, and restricted subspecialty coverage. Workforce planning should therefore incorporate not only the number of specialists trained but also retention, workload, professional well-being, mentorship, and opportunities for academic development.

Another challenge for Costa Rican neurosurgery is its academic output. Although Costa Rica has a relatively strong scientific presence within Central America, this broader research productivity has not been similarly reflected in neurosurgery. Costa Rica, together with Panama and Guatemala, accounts for approximately 82% of the region's indexed scientific output (44, 45). In contrast, Brazil, Mexico, Argentina, and Colombia account for 76% of the neurosurgical academic output; Costa Rica was not among the leading contributors to the regional literature (41). This discrepancy is also reflected in the sources identified in the present review. Much of the historical record of Costa Rican neurosurgery is preserved in institutional archives, hospital historical reviews, anniversary publications, and other forms of grey literature rather than in indexed, peer-reviewed journals. Thus, despite a relatively mature clinical neurosurgical infrastructure, Costa Rica appears to have developed a less substantial specialty-specific research and publication profile. Strengthening local research capacity, institutional leadership, and opportunities for collaboration may therefore represent an important component of the continued development of Costa Rican neurosurgery.

### Limitations

This review has several limitations inherent to its historical and descriptive design. First, it does not follow a systematic search methodology; although a structured, multi-source approach was used (see Methods), a formal systematic or scoping review protocol could identify additional relevant sources. Second, the manuscript relies substantially on institutional archives, anniversary publications, and other non-peer-reviewed historical material; such sources are subject to selection and reporting bias, particularly regarding the attribution of institutional “firsts” and the recollection of events that occurred several decades ago. Third, some historical claims could only be corroborated by a single source. Fourth, contemporary workforce and demographic estimates are dynamic figures that change over time as new specialists are registered or emigrate; the figures reported here reflect the most recent registry data available at the time of writing (2025-2026) and should be interpreted as a snapshot rather than a fixed value. Finally, as a single-country historical narrative, this review cannot fully capture the diversity of institutional experiences across all regional and peripheral facilities in Costa Rica, and its regional comparisons are necessarily selective rather than exhaustive.

## Conclusion

The development of neurosurgery in Costa Rica reflects the broader evolution of specialized medicine in Latin America, shaped by the expansion of public healthcare institutions, academic training, and international collaboration. From its early origins within general surgery to its formal consolidation, the specialty has developed into a structured national service capable of providing advanced neurosurgical care. Despite this progress, challenges such as workforce limitations, nearly absent academic output, and the absence of domestic subspecialty training remain. Continued investment in training and international collaborations, including symmetric academic partnerships, will be important for sustaining the future growth of neurosurgery in the country.
